# To a Future Where Everyone Can Walk a Dog Even if They Don't Own One

**DOI:** 10.3389/fpubh.2018.00349

**Published:** 2018-11-30

**Authors:** Eunice Y. Chen

**Affiliations:** Department of Psychology, Temple University, Philadelphia, PA, United States

**Keywords:** animals, dogs, walking, overweight, students, humans, animal-assisted therapy

Despite desperately wanting a dog, like many children, because of restricted financial circumstances, I did not have the good fortune of owning a dog as a child. However, research may overcome this barrier so that lack of dog ownership need not be a barrier to spending time with and walking dogs.

The US Department of Health and Human Services recommends walking 5 days a week for at least 45 minutes at a time, with 30 minutes at a moderate to brisk pace of 3–4 miles per hour and 15 minutes at a very brisk walking of 5–6 miles per hour (1). This level of physical activity is associated with a decreased risk of mortality, cardiorespiratory disease, and increased likelihood for weight-loss, and improvements in musculoskeletal health (2). Only about half of Americans engage in this recommended amount of physical activity (3). But if every American had a healthy dog and walked it regularly, they would be more likely to achieve these recommended levels of activity (4).

Multiple studies show that dog ownership improves human health. Becoming a dog owner increases physical activity (4–7) and walking (8–10), reduces your weight (11), and decreases your odds of diabetes, hypertension, hypercholesterolemia, and depression (12–15). Dog ownership reduces predictors of negative cardiovascular outcomes like blood pressure (15–19), triglycerides (20, 21), and stress (22–25). Indeed, owning a dog increases the likelihood of you surviving a heart attack (26–28) such that even the American Heart Association advocates dog ownership as a way to reduce the risk of cardiovascular disease (29). Australian, German, and Chinese studies show that pet ownership decreases doctor visits, and reduces the likelihood of cardiac problems and sleeping difficulties (30–33). Interventions with dogs improve the outcome of children, adolescents, and adults with a range of medical and psychological problems including post-traumatic stress disorder, developmental disabilities, schizophrenia, autism-spectrum disorders, and cancer (34–38). In a short period of time, human-animal studies have progressed from small experimental studies to studies assessing the public health impact of dogs on human lives, including increasing human physical activity, please see these reviews (6, 34–43).

Despite the double challenge of conducting clinical trials with humans and animals (44) randomized controlled trials are needed in this field. For instance in considering the needs of both dogs and humans, human-animal trials require approval from both human and animal institutional review boards. However, the randomized controlled trial is the gold standard for the assessment of intervention efficacy because it most effectively and efficiently evaluates an intervention's effect, eliminating systematic, and random bias (45).

A search using the terms “randomized controlled trial” AND “dogs” and “walking” resulted in 40 hits in PubMed up to 4/15/2018. When studies with groups with psychological or medical problems were excluded, this yielded five randomized controlled trials (46–51), that examine the effects of dog-walking on human walking, see Table 1. An effect size could not be calculated for one of the five randomized controlled trials, and of the remaining 4 randomized controlled trials, two had moderate to large effects (Hedges g) for the dog-walking intervention arm, and two of the four had small effects.

**Table 1:** Randomized controlled trials examining the effect of dog-walking interventions on human walking.

**Study**	**Intervention**	**Physical activity**	**Assesed at # months**	***n/N***	***g***
Richards et al. (46)	3 month intervention: (1) Dog owners + intervention (weekly emails addressing self-efficacy, social support, goal setting, and benefits/barriers to walking). Other arms were (2) Non-dog owners + intervention, (3) Non-dog owners with control intervention (emailed Physical activity guidelines) intervention and (4) Dog owners with control intervention.	Min/wk	6	20/65	1.00
Schneider et al. (47)	6 month intervention: (1) Dog owners + social online network meetup and newsletters. Other arm was (2) Dog owners + control intervention (emailed physical activity guidelines)	Steps/day	6	45/102	0.21
Rhodes et al. (48)	3 month intervention: (1) Dog owners+ Persuasive information about dog health and walking, and walking calendar. Other arm was (2) Dog owners walking-as-usual	Min/wk	3	30/58	0.72
Morrison et al. (49)	2.5 month intervention: (1) Dog owners and their families + behavioral intervention. Other arm was (2) Dog owners and families with no intervention.	ParentActigraph counts/min/wk	2.5	15/27	0.15
Byers et al. (50, 51)	1 week intervention: (1) Dog owners and their overweight dogs + vets physical activity prescription. Other arm was (2) Dog owners and their overweight dogs + standard care	Human steps unreported.	3	32/72	NA

Calculation for Hedges g = (m_1_ −m_2_)/s^*^ where m_1_ = baseline mean, m_2_ = mean at 2nd timepoint, s^*^ = √[(n_1_−1)s_1_^2^ + (n_2_−1) s_2_^2^ / (n_1_ +n_2_−2)] where n_1_ = baseline sample size, n_2_ is sample size at 2nd timepoint calculated on the intervention arm of interest. In Table 1, n = number of participants in intervention arm of interest, N = number of participants in the whole study. NA = not available.

These randomized controlled trials focus on dog owners walking their dogs. However, randomized controlled trials that focus on dog owners walking their own dogs may limit whether dog-walking interventions can be “scaled-up” or implemented on a more widespread basis (52).

Certain correlates may distinguish dog owners from non-dog owners. For instance, a large US study showed that dog ownership is associated with being white, with home ownership, and with living in a house (53). A review of the literature also suggests that living close to places wheredogs can be walked (41) also increases the likelihood of owning a dog. The implication of this is that racial diversity, renting rather than owning a home, living in an apartment, and possibly socioeconomic disadvantage may decrease the likelihood of dog ownership. So although 44% of households in the US (2015–2016) are estimated to own a dog, the majority of households do not (54).

But does lack of dog ownership have to be a barrier to dog-walking interventions? One of the foreseeable challenges for dog-walking interventions targeting human physical activity is working out how to scale this intervention to individuals who do not own a dog. There are numerous online media reports of shelter dog-walking programs, even phone applications for walking shelter dogs. However, trials published in peer-reviewed journals are scant (55, 56). One small open trial with public housing residents showed that overweight individuals who borrowed and walked dogs from a dog-shelter had small (hedges g = 0.17), but significant weight loss (57). This suggests that pairing individuals who do not own a dog with dogs in rescues or shelters may be a feasible weight loss solution.

Designing behavioral interventions that can “scale-up” is increasingly becoming an important criteria for the success of an intervention (58, 59). One of the ways that this critical barrier can be addressed is by considering mutually beneficial partnerships between dog shelters/rescues and other institutions, some of which may seem improbable at first.

An important start is to take into account both the socio-ecological structure and function of institutions. Drawing on Bronferonner's work (60), Westgarth et al. (41) describes the structure of a socio-ecological model of dog-walking that highlights the individual sphere of influence and its dog-related factors, as well as more distal social-environmental, and physical-environmental factors that are associated with dog-walking (6). This can add to a consideration of the functions of different institutions and how these can promote healthy behaviors in both individuals and institutions.

Human-dog relationships have been described as a form of social capital which is defined as an “investment in social relations with expected returns” (61–63). On a system-level mutually reinforcing, sustainable partnerships can be formed between institutions to improve the health of both humans and animals (62, 63). Social capital requires the utilization of resources embedded in a social structure, accessibility to this, and the mobilization of these resources for purposive action, e.g., improved health for both humans and animals (62, 63). Increasing physical activityis a serious public health challenge; as is ensuring that homeless dogs are cared for. Considering a model of human-dog interventions that considers function as well as the structure of institutions and individuals is empowering and self-sustaining, and has the potential to build scalable, sustainable interventions.

Everyone who does not own a dog could probably benefit from walking a dog daily. However, there may be certain institutions that could provide the other half of a mutually beneficial partnership. For instance, with appropriate supervision, various institutions could offer dog-walking opportunities: health care institutions like rehabilitation centers, half-way houses, group homes or elder care facilities; educational institutions, such as colleges or schools; even insurance companies.

Is it possible to do something like this? Recently undergraduates at Temple University undertook this endeavor. Temple University is a large urban college in Center City Philadelphia consisting of a socioeconomically and racially diverse group of more than 30,000 undergraduates. After a review of the literature (64) over the summer of 2017, one of my lab undergraduates sent an informational Facebook message on July 18th, 2017 to the incoming freshmen class and in one day 22 incoming freshmen posted their interest (and photos of their dogs) in joining a volunteer dog-walking association, with another 56 freshmen signing up. In 10 days, 172 incoming freshmen posted their interest in joining the volunteer dog-walking association. On December 6th, 2017, the “Diamond Dogs” undergraduate dog-walking association was formally ratified by the university, with support from the Dean's office and since then there have been 3 townhall meetings of ~70 students a time. “Diamond Dogs” is partnered with two inner-city dog rescues approximately a mile from Temple University's campus. My last communication with the rescues was that students are participating in their orientations and training and are walking their dogs. From the perspective of dog rescues, this is a “win-win.” Inner city rescues in Philadelphia find it difficult to recuit regular dog-walkers but a schedule of regular volunteer walkers has eased their need to play and to walk their healthy dogs daily.

Humans form strong attachments to pets, particularly dogs (65–68) and pets increase social capital through creating more social connections and networks (61, 69, 70). But will people who don't own a dog develop an attachment for a dog at a shelter or rescue who may leave in a couple of weeks because they have been adopted? Are the positive cardiovascular effects in response to stressful situations also found in non-dog owners who interact with shelter or rescue dogs (71)? Can shelter/rescue dog-walking interventions increase the likelihood of walkers to eventually adopt a dog? Can cortisol or immunological measures be used to check that shelter/rescue dogs are experiencing less stress with regular walkers (72)? While these are important research questions there are also important ethical and practical issues to consider in research of this nature.

Rescue and shelter dogs often have greater psychological and medical needs than dogs not in a shelter or rescue. Volunteers at shelters are likely to be pet-owners (73) and shelters and rescues are likely to prefer experienced dog owners rather than non-dog owners to walk their dogs. Guidelines from the Association of Shelter Veterinarians (74), and from the American Veterinary Medical Association (75) and the Humane Society (76) highlight the importance of training and supervising shelter and rescue volunteers, including basic training in animal handling and bite prevention. However, research addressing the training of non-dog owner volunteers is in its infancy (77, 78). Some questions that will need to be answered include: how much training do non-dog owner volunteer walkers need to achieve the skill level of experienced dog owners? How are these skills best assessed? And what are the effects of volunteers with varied experience on shelter dogs?

Assessing the efficacy of shelter dog-walking intervention for non- owners, deserves the best designs and methods. This means that non-peer reviewed reports by the media are not sufficient as evidence for the efficacy of interventions like this and the use of phone applications to encourage the walking of shelter dogs warrant thoughtful and rigorous testing prior to widespread use or claims about their effect. Clinical trials with shelter dogs and humans need to meet both Human Institutional Review Board and Institutional Animal Care and Use Committee requirements. The design and oversight of these trials require the partnership of human clinical trial experts, human-animal intervention research experts, veternarians, and shelters/rescues. There are not only ethical issues that must be considered but also legal issues in clinical trials of this nature. For instance animal rescues in the United States are governed by state, county and city ordinances that may differ between rescues. Careful attention to these ordinances are needed in rescue dog-walking intervention trials.

It is important to conduct clinical trials to test if it is possible to engage educational and social institutions in mutually reinforcing partnerships to improve the health of people and animals. The Shelter dog-walking intervention proposed can potentially make dog-walking a scalable intervention and has broad applicability to a wide variety of institutions and partnerships.

Despite the challenges, I'm looking forward to a future where everyone can walk a dog even if they don't own one.

## Author contributions

EC conceived of this idea and opinion and wrote this paper.

### Conflict of interest statement

The author declares that the research was conducted in the absence of any commercial or financial relationships that could be construed as a potential conflict of interest.

## References

[1] United States Department of Health and Human Services 2018 Physical Activity Guidelines Advisory Committee Scientific Report (2018). Available online at: https://health.gov/paguidelines/second-edition/report/ (Accessed November 20, 2018).

[2] American College of Sports Medicine's Guidelines for Exercise Testing and Prescription 10th ed. China: Wolters Kluwer (2018).

[3] Clarke TC Norris T Schiller JS Early Release of Selected Estimates Based on Data from the 2016 National Health Interview Survey. National Center for Health Statistics (2017). Available online at: https://www.cdc.gov/nchs/nhis/releases/released201705.htm#TechNotes

[4] Soares J Epping JN Owens CJ Brown DR Lankford TJ Simoes EJ. Odds of getting adequate physical activity by dog walking. J Phys Activity Health (2015) 12:S102–S9. 24733365 10.1123/jpah.2013-0229 PMC4535333

[5] Yabroff KR Troiano RP Berrigan D. Walking the dog: is pet ownership associated with physical activity in California? J Phys Activity Health (2008) 5:216–28. 10.1123/jpah.5.2.216 18382031

[6] Christian HE Westgarth C Bauman A Richards EA Rhodes RE Evenson KR. Dog ownership and physical activity: a review of the evidence. J Phys Activity Health (2013) 10:750–9. 10.1123/jpah.10.5.750 23006510

[7] Serpell J. Beneficial effects of pet ownership on some aspects of human health and behaviour. J R Soc Med. (1991) 84:717. 10.1177/014107689108401208 1774745 PMC1295517

[8] Cutt HE Knuiman MW Giles-Corti B. Does getting a dog increase recreational walking? Int J Behav Nutr Phys Activity (2008) 5:17. 10.1186/1479-5868-5-17 18366804 PMC2359767

[9] Oka K Shibata A. Dog ownership and health-related physical activity among Japanese adults. J Phys Activity Health (2009) 6:412–8. 10.1123/jpah.6.4.412 19842454

[10] Christian H Wood L Nathan A Kawachi I Houghton S Martin K. The association between dog walking, physical activity and owner's perceptions of safety: cross-sectional evidence from the US and Australia. BMC Public Health (2016) 16:1010. 10.1186/s12889-016-3659-8 27658384 PMC5034524

[11] Coleman KJ Rosenberg DE Conway TL Sallis JF Saelens BE Frank LD. Physical activity, weight status, and neighborhood characteristics of dog walkers. Prevent Med. (2008) 47:309–12. 10.1016/j.ypmed.2008.05.007 18572234 PMC2587031

[12] Lentino C Visek AJ McDonnell K DiPietro L. Dog walking is associated with a favorable risk profile independent of moderate to high volume of physical activity. J Phys Activity Health (2012) 9:414–20. 10.1123/jpah.9.3.414 21934154

[13] Aiba N Hotta K Yokoyama M Wang G Tabata M Kamiya K Usefulness of pet ownership as a modulator of cardiac autonomic imbalance in patients with diabetes mellitus, hypertension, and/or hyperlipidemia. Am J Cardiol. (2012) 109:1164–70. 10.1016/j.amjcard.2011.11.055 22277896

[14] Weber KS Roden M Mussig K. Do dogs sense hypoglycaemia? Diabet Med. (2016) 33:934–8. 10.1111/dme.12975 26433211

[15] Anderson WP Reid CM Jennings GL. Pet ownership and risk factors for cardiovascular disease. Med J Austr. (1992) 157:298–301. 1435469

[16] Chowdhury EK Nelson MR Jennings GL Wing LM Reid CM Committee AM. Pet ownership and survival in the elderly hypertensive population. J Hypertension (2017) 35:769–75. 10.1097/HJH.0000000000001214 28009706

[17] Grossberg JM Alf EF Interaction with pet dogs: effects on human cardiovascular response. J Delta Soc. (1985) 2:20–7.

[18] Jenkins JL. Physiological effects of petting a companion animal. Psychol Rep. (1986) 58:21–2. 10.2466/pr0.1986.58.1.21 3961065

[19] Allen K Blascovich J Mendes WB. Cardiovascular reactivity and the presence of pets, friends, and spouses: the truth about cats and dogs. Psychosom Med. (2002) 64:727–39. 10.1097/01.PSY.0000024236.11538.41 12271103

[20] Dembicki D Anderson J. Pet ownership may be a factor in improved health of the elderly. J Nutr Elderly (1996) 15:15–31. 10.1300/J052v15n03_02 8948954

[21] Schreiner PJ. Emerging cardiovascular risk research: impact of pets on cardiovascular risk prevention. Curr Cardiovasc Risk Rep. (2016) 10:8. 10.1007/s12170-016-0489-2 27547289 PMC4991891

[22] Barker SB Knisely JS McCain NL Best AM. Measuring stress and immune response in healthcare professionals following interaction with a therapy dog: A pilot study. Psychol Rep. (2005) 96:713–29. 10.2466/pr0.96.3.713-729 16050629

[23] Beetz A Kotrschal K Turner DC Hediger K Uvnäs-Moberg K Julius H The effect of a real dog, toy dog and friendly person on insecurely attached children during a stressful task: an exploratory study. Anthrozoös (2011) 24:349–68. 10.2752/175303711X13159027359746

[24] Odendaal J Animal-assisted therapy—magic or medicine? J Psychosomat Res. (2000) 49:275–80. 10.1016/S0022-3999(00)00183-5 11119784

[25] Krause-Parello CA Tychowski J Gonzalez A Boyd Z. Human–canine interaction: exploring stress indicator response patterns of salivary cortisol and immunoglobulin A. Res Theory Nurs Pract. (2012) 26:25–40. 10.1891/1541-6577.26.1.25 22866562

[26] Friedmann E Katcher AH Lynch JJ Thomas SA. Animal companions and one-year survival of patients after discharge from a coronary care unit. Public Health Rep. (1980) 95:307. 6999524 PMC1422527

[27] Friedmann E Thomas SA. Pet ownership, social support, and one-year survival after acute myocardial infarction in the Cardiac Arrhythmia Suppression Trial (CAST). Am J Cardiol. (1995) 76:1213–7. 10.1016/S0002-9149(99)80343-9 7502998

[28] Friedmann E Thomas SA Son H. Pets, depression and long-term survival in community living patients following myocardial infarction. Anthrozoös (2011) 24:273–85. 10.2752/175303711X13045914865268 21857770 PMC3156485

[29] Levine GN Allen K Braun LT Christian HE Friedmann E Taubert KA. Pet ownership and cardiovascular risk: a scientific statement from the American Heart Association. Circulation (2013) 127:2353–63. 10.1161/CIR.0b013e31829201e1 23661721

[30] Headey B Health benefits and health cost savings due to pets: preliminary estimates from an Australian national survey. Soc Indic Res. (1999) 47:233–43. 10.1023/A:1006892908532

[31] Headey B Grabka M Kelley J Reddy P Tseng Y-P Pet ownership is good for your health and saves public expenditure too: Australian and German longitudinal evidence. Austr Soc Monitor (2002) 5:93–9. Available online at: https://search.informit.com.au/documentSummary;dn=674270738133649;res=IELBUS

[32] Headey B Grabka MM Pets and human health in Germany and Australia: National longitudinal results. Soc Indic Res. (2007) 80:297–311. 10.1007/s11205-005-5072-z

[33] Headey B Na F Zheng R Pet dogs benefit owners' health: a ‘natural experiment’ in China. Soc Indic Res. (2008) 87:481–93. 10.1007/sl1205-007-9142-2

[34] Nimer J Lundahl B Animal-assisted therapy: a meta-analysis. Anthrozoös (2007) 20:225–38. 10.2752/089279307X224773

[35] Brelsford VL Meints K Gee NR Pfeffer K. Animal-Assisted Interventions in the Classroom—a systematic review. Int J Environ Res Public Health (2017) 14:669. 10.3390/ijerph14070669 28640200 PMC5551107

[36] Maujean A Pepping CA Kendall E A systematic review of randomized controlled trials of animal-assisted therapy on psychosocial outcomes. Anthrozoös (2015) 28:23–36. 10.2752/089279315X14129350721812

[37] Hoagwood KE Acri M Morrissey M Peth-Pierce R Animal-assisted therapies for youth with or at risk for mental health problems: a systematic review. Appl Dev Sci. (2017) 21:1–13. 10.1080/10888691.2015.1134267 28798541 PMC5546745

[38] Gee NR Griffin JA McCardle P Human–animal interaction research in school settings: current knowledge and future directions. AERA Open (2017) 3:2332858417724346 10.1177/2332858417724346

[39] Christian H Bauman A Epping JN Levine GN McCormack G Rhodes RE. Encouraging dog walking for health promotion and disease prevention. Am J Lifestyle Med. (2016) 12:233–43. 10.1177/1559827616643686 30202393 PMC6124971

[40] Purewal R Christley R Kordas K Joinson C Meints K Gee N. Companion animals and child/adolescent development: a systematic review of the evidence. Int J Environ Res Public Health (2017) 14:234. 10.3390/ijerph14030234 28264460 PMC5369070

[41] Westgarth C Christley RM Christian HE. How might we increase physical activity through dog walking?: a comprehensive review of dog walking correlates. Int J Behav Nutr Phys Act. (2014) 11:83. 10.1186/1479-5868-11-83 25142228 PMC4261546

[42] Arhant-Sudhir K Arhant-Sudhir R Sudhir K. Pet ownership and cardiovascular risk reduction: supporting evidence, conflicting data and underlying mechanisms. Clin Exp Pharmacol Physiol. (2011) 38:734–8. 10.1111/j.1440-1681.2011.05583.x 21824172

[43] McCune S Kruger KA Griffin JA Esposito L Freund LS Hurley KJ Evolution of research into the mutual benefits of human–animal interaction. Anim Front. (2014) 4:49–58. 10.2527/af.2014-0022

[44] Serpell J McCune S Gee N Griffin JA Current challenges to research on animal-assisted interventions. Appl Dev Sci. (2017) 21:223–33. 10.1080/10888691.2016.1262775

[45] American Psychological Association Criteria for evaluating treatment guidelines. Am Psychol. (2002) 57:1052–9. 10.1037/0003-066X.57.12.1052 12617064

[46] Richards EA Ogata N Cheng C-W Randomized controlled theory-based, e-mail-mediated walking intervention: differences between dog owners and non-dog owners. Clin Nurs Res. (2017) 26:47–67. 10.1177/1054773816657799 27369044

[47] Schneider KL Murphy D Ferrara C Oleski J Panza E Savage C. An online social network to increase walking in dog owners: a randomized trial. Med Sci Sports Exercise (2015) 47:631. 10.1249/MSS.0000000000000441 25003777 PMC4286532

[48] Rhodes RE Murray H Temple VA Tuokko H Higgins JW. Pilot study of a dog walking randomized intervention: effects of a focus on canine exercise. Prevent Med. (2012) 54:309–12. 10.1016/j.ypmed.2012.02.014 22405707

[49] Morrison R Reilly JJ Penpraze V Westgarth C Ward DS Mutrie N. Children, parents and pets exercising together (CPET): exploratory randomised controlled trial. BMC Public Health (2013) 13:1096. 10.1186/1471-2458-13-1096 24279294 PMC4222564

[50] Byers CG Wilson CC Stephens MB Goodie JL Netting FE Olsen CH Owners and pets exercising together: Canine response to veterinarian-prescribed physical activity. Anthrozoös (2014) 27:325–33. 10.2752/175303714X14036956449224

[51] Stephens MB Wilson CC Goodie JL Netting FE Olsen CH Byers CG. Health perceptions and levels of attachment: owners and pets exercising together. J Am Board Family Med. (2012) 25:923–6. 10.3122/jabfm.2012.06.110325 23136334

[52] Milat AJ King L Bauman AE Redman S. The concept of scalability: increasing the scale and potential adoption of health promotion interventions into policy and practice. Health Promot Int. (2012) 28:285–98. 10.1093/heapro/dar097 22241853

[53] Saunders J Parast L Babey SH Miles JV. Exploring the differences between pet and non-pet owners: implications for human-animal interaction research and policy. PloS ONE (2017) 12:e0179494. 10.1371/journal.pone.0179494 28644848 PMC5482437

[54] APPA American Pets Association National Pet Owners Survey (2016).

[55] Gunter L Protopopova A Hooker SP Der Ananian C Wynne CDL. Impacts of encouraging dog walking on returns of newly adopted dogs to a shelter. J Appl Anim Welfare Sci. (2017) 20:357–71. 10.1080/10888705.2017.1341318 28696774

[56] Bright TM Hadden L. Safewalk: improving enrichment and adoption rates for shelter dogs by changing human behavior. J Appl Anim Welfare Sci. (2017) 20:95–105. 10.1080/10888705.2016.1247353 27827535

[57] Johnson RA Meadows RL. Dog-walking: motivation for adherence to a walking program. Clin Nurs Res. (2010) 19:387–402. 10.1177/1054773810373122 20651066

[58] Reis RS Salvo D Ogilvie D Lambert EV Goenka S Brownson RC. Scaling up physical activity interventions worldwide: stepping up to larger and smarter approaches to get people moving. Lancet (2016) 388:1337–48. 10.1016/S0140-6736(16)30728-0 27475273 PMC5193005

[59] Lutz W. Global sustainable development priorities 500 y after luther: Sola schola et sanitate. Proc Natl Acad Sci USA. (2017) 114:6904–13. 10.1073/pnas.1702609114 28630291 PMC5502620

[60] Bronfenbrenner U The Ecology of Human Development. Cambridge, MA: Harvard University Press (1979).

[61] Lin N Building a network theory of social capital. Connections (1999) 22:28–51.

[62] Wood L Giles-Corti B Bulsara M. The pet connection: pets as a conduit for social capital? Soc Sci Med. (2005) 61:1159–73. 10.1016/j.socscimed.2005.01.017 15970228

[63] Lin N Cook KS Burt RS Social Capital: Theory and Research. New York, NY: Routledge (2017).

[64] Chen E Murray S Arlt J Middleton D Conklin C Windom CAA Systematic review of RCTs for Dog-walking and Pilot Study with Fitbark & Fitbit M-Health Devices. The Obesity Society: Obesity Week 2017. Washington, DC (2017). Available online at: https://higherlogicdownload.s3.amazonaws.com/OBESITY/004d4f70-37d5-434e-b24d-08a32dfdfcd9/UploadedImages/2017ow_abstracts/11-2-Thursday-LB-Poster-Abstracts.pdf (Accessed November 20, 2018).

[65] Archer J Why do people love their pets? Evol Hum Behav. (1997) 18:237–59. 10.1016/S0162-3095(99)80001-4

[66] Stallones L Marx MB Garrity TF Johnson TP Pet ownership and attachment in relation to the health of US adults, 21 to 64 years of age. Anthrozoös (1990) 4:100–12. 10.2752/089279391787057206

[67] Marx MB Stallones LB Garrity TF Johnson TP Demographics of pet ownership among U.S. adults 21 to 64 years of age. Anthrozoös (1988) 2:33–7. 10.2752/089279389787058262

[68] Zasloff RL Measuring attachment to companion animals: a dog is not a cat is not a bird. Appl Anim Behav Sci. (1996) 47:43–8. 10.1016/0168-1591(95)01009-2

[69] Wood LJ Giles-Corti B Bulsara MK Bosch DA More than a furry companion: the ripple effect of companion animals on neighborhood interactions and sense of community. Soc Anim. (2007) 15:43–56. 10.1163/156853007X169333

[70] Wood L Martin K Christian H Nathan A Lauritsen C Houghton S. The pet factor–companion animals as a conduit for getting to know people, friendship formation and social support. PLoS ONE (2015) 10:e0122085. 10.1371/journal.pone.0122085 25924013 PMC4414420

[71] Kingwell BA Lomdahl A Anderson WP. Presence of a pet dog and human cardiovascular responses to mild mental stress. Clin Autonom Res. (2001) 11:313–7. 10.1007/BF02332977 11758798

[72] Dreschel N Granger D Advancing the social neuroscience of human-animal interaction: the role of salivary bioscience. In: Freund L, McCardle P, Esposito L, Griffin J, editors. The Social Neuroscience of Human-Animal Interaction Washington, DC: American Psychological Association (2016). p. 210–30. 10.1037/14856-001

[73] Neumann SL Animal welfare volunteers: who are they and why do they do what they do? Anthrozoös (2010) 23:351–64. 10.2752/175303710X12750451259372

[74] Newbury S Blinn MK Bushby PA Cox CB Dinnage JD Griffin B Guidelines for Standards of Care in Animal Shelters. Association of Shelter Veterinarians (2010), 1–64. Available online at: https://www.sheltervet.org/assets/docs/shelter-standards-oct2011-wforward.pdf (Accessed November 20, 2018).

[75] American Veterinary Medical Association Guidelines for Animal Assisted Activity, Animal-Assisted Therapy and Resident Animal Programs (2009). Schaumburg, IL: American Veterinary Medical Association.

[76] McFarland B Volunteer Management for Animal Care Organizations. 2nd ed. The Humane Society of the United States. Washington, DC: Human Society Press (2005).

[77] Howard VJ DiGennaro Reed FD. Training shelter volunteers to teach dog compliance. J Appl Behav Anal. (2014) 47:344–59. 10.1002/jaba.120 24924218

[78] Howard VJ Digennaro Reed FD An evaluation of training procedures for animal shelter volunteers. J Organ Behav Manage. (2015) 35:296–320. 10.1080/01608061.2015.1093052

